# Osmoregulatory bicarbonate secretion exploits H^+^-sensitive haemoglobins to autoregulate intestinal O_2_ delivery in euryhaline teleosts

**DOI:** 10.1007/s00360-014-0844-x

**Published:** 2014-08-27

**Authors:** C. A. Cooper, M. D. Regan, C. J. Brauner, E. S. R. De Bastos, R. W. Wilson

**Affiliations:** 1 Department of Chemistry, Wilfrid Laurier University, 75 University Ave. West, Waterloo, ON N2L 3C5 Canada; 2 Department of Zoology, University of British Columbia, 6270 University Boulevard, Vancouver, BC Canada; 3 Biosciences, College of Life and Environmental Sciences, University of Exeter, Geoffrey Pope Building, Exeter, Devon EX4 4QD UK

**Keywords:** NTP, Haemoglobin, Buffer values, Hypersalinity, Bicarbonate secretion, Haldane effect, Bohr effect, Proton

## Abstract

Marine teleost fish secrete bicarbonate (HCO_3_^−^) into the intestine to aid osmoregulation and limit Ca^2+^ uptake by carbonate precipitation. Intestinal HCO_3_^−^ secretion is associated with an equimolar transport of protons (H^+^) into the blood, both being proportional to environmental salinity. We hypothesized that the H^+^-sensitive haemoglobin (Hb) system of seawater teleosts could be exploited via the Bohr and/or Root effects (reduced Hb-O_2_ affinity and/or capacity with decreasing pH) to improve O_2_ delivery to intestinal cells during high metabolic demand associated with osmoregulation. To test this, we characterized H^+^ equilibria and gas exchange properties of European flounder (*Platichthys flesus*) haemoglobin and constructed a model incorporating these values, intestinal blood flow rates and arterial–venous acidification at three different environmental salinities (33, 60 and 90). The model suggested red blood cell pH (pH_i_) during passage through intestinal capillaries could be reduced by 0.14–0.33 units (depending on external salinity) which is sufficient to activate the Bohr effect (Bohr coefficient of −0.63), and perhaps even the Root effect, and enhance tissue O_2_ delivery by up to 42 % without changing blood flow. In vivo measurements of intestinal venous blood pH were not possible in flounder but were in seawater-acclimated rainbow trout which confirmed a blood acidification of no less than 0.2 units (equivalent to −0.12 for pH_i_). When using trout-specific values for the model variables, predicted values were consistent with measured in vivo values, further supporting the model. Thus this system is an elegant example of autoregulation: as the need for costly osmoregulatory processes (including HCO_3_^−^ secretion) increases at higher environmental salinity, so does the enhancement of O_2_ delivery to the intestine via a localized acidosis and the Bohr (and possibly Root) effect.

## Introduction

The marine teleost intestine is a multifunctional organ. It is an important site of digestion, nutrient absorption and metabolic waste excretion in all fishes (Taylor et al. 2011), and in marine teleosts, the intestine is also vital to the absorption of water and excretion of divalent ions (Wilson et al. 2002; Wilson and Grosell 2003). Calcium and magnesium ions in particular are ingested with seawater and, if not dealt with, could accumulate to detrimental levels. This is prevented by their precipitation in carbonate crystals through the secretion of metabolically produced bicarbonate (HCO_3_^−^) into the intestine from epithelial cells lining the lumen (Cooper et al. 2010; Walsh et al. 1991; Wilson et al. 2002; Grosell, 2006, 2011; Wilson and Grosell, 2003; Whittamore et al. 2010).

Although essential to osmoregulation, intestinal HCO_3_^−^ secretion also affects the acid–base balance of marine teleosts. The production of HCO_3_^−^ from endogenous carbon dioxide (CO_2_) liberates a proton (H^+^) that, in order to maintain the intestinal cell’s pH, is extruded basolaterally into the blood (Genz et al. 2008; Grosell et al. 2005; Grosell and Genz 2006; Wilson et al. 2002; Wilson and Grosell 2003) via a sodium-dependent pathway and possible proton pumps (Grosell et al. 2009a, 2009b; Grosell 2010, 2011). Alternatively, the source of HCO_3_^−^ in some species or regions of the intestine is transepithelial transfer across both basolateral and apical membranes. However, for either mechanism, the overall effect is quantitatively identical, i.e. a significant blood acidosis in proportion to net HCO_3_^−^ secretion rates which are particularly high during enhanced Ca^2+^ supply to the gut associated with living in hypersaline waters (Genz et al. 2008) or experimental intestinal perfusion with high Ca^2+^ solutions (Cooper et al. 2010; Whittamore et al. 2010). Furthermore, intestinal HCO_3_^−^ production comes with a metabolic cost, as enhanced HCO_3_^−^ secretion after a meal has been shown to almost double O_2_ consumption in the intestinal tissue of gulf toadfish (*Opsanus beta*) in vitro (Taylor and Grosell 2009). In a situation of high demand for HCO_3_^−^ secretion into the intestinal lumen, a mechanism to promote O_2_ delivery would be beneficial.

Teleost haemoglobins (Hbs) are particularly sensitive to protons, exhibiting some of the largest Bohr effects (reduction in Hb-O_2_ affinity with a pH reduction) observed in the animal kingdom (Berenbrink et al. 2005). Furthermore, teleost Hbs also exhibit a Root effect, where a reduction in blood pH results in a reduction in blood O_2_ carrying capacity (Root 1931; Root and Irving 1943). Provided blood pH is reduced during arterial–venous blood transit, these pH-sensitive Hbs allow for enhanced O_2_ delivery to the tissues. Under steady-state aerobic metabolism, the reduction in blood pH during arterial–venous blood transit arises primarily from metabolically produced CO_2_. Tissue respiratory quotients (RQ; CO_2_ produced relative to O_2_ consumed) are typically 0.7–1.0, and the optimal Bohr coefficient for O_2_ delivery has been calculated to be ½ of RQ (i.e. a Bohr coefficient of −0.35 to −0.5) (Lapennas 1983), but most teleost fishes have Bohr coefficients in excess of this value (Berenbrink et al. 2005) which could result in an impairment in O_2_ delivery. Interestingly, in many teleosts with a large Bohr coefficient, the magnitude of the Bohr coefficient is non-linear over different regions of the oxygen equilibrium curve and may be reduced close to this optimal value during some physiological states such as exercise (Brauner et al. 2000b). However, in tissues where arterial–venous blood transit experiences extra acidification in addition to that associated with aerobic metabolism, enhanced O_2_ delivery can occur via the Bohr/Root effect regardless of the magnitude of the Bohr coefficient. This is the case in the teleost swim bladder and eye, where exceptionally high arterial partial pressures of O_2_ (*P*O_2_) are needed to drive O_2_ over large diffusion distances and against high hydrostatic pressures (Pelster and Randall 1998).

We hypothesized that a similar O_2_ delivery mechanism may also take place at the intestine of marine teleosts, where the additional blood acidification associated with secretion of HCO_3_^−^ for Ca^2+^ and Mg^2+^ precipitation in the gut may exploit a Bohr/Root effect and enhance O_2_ delivery. It follows that the magnitude of this effect on O_2_ delivery would increase with salinity. To test these hypotheses, we acclimated European flounder (*Platichthys flesus*), a species with a well-resolved HCO_3_^−^ secretion mechanism, to a number of salinities with the objective of characterizing the Bohr/Root effects of their Hb system. To ascertain this, further experiments were required to estimate the arterial–venous pH change in the capillaries supplying the intestinal cells. As this proved impossible in flounder, in vivo measurements of intestinal venous blood pH using seawater-acclimated rainbow trout (*Oncorhynchus mykiss*) were performed. From this, it was possible to understand how those Bohr/Root effects might be used to maximize O_2_ delivery to the intestine during osmoregulation in hyperosmotic environments.

## Methods

### Fish husbandry

Flounder (*Platichthys flesus*) (400 ± 64 g) were obtained from Flookburgh, Cumbria, UK. Prior to experiments, fish were maintained in 150-l aerated flow through tanks containing artificial sea water at a salinity of 33 (Tropic Marin Sea Salt, Tropic Marin Centre, UK, added to deionised water) and 12 °C, under a 12-h light:dark photoperiod. Fish were fed weekly on ragworm (*Nereis virens*), although they were not fed for at least 72 h before experimentation. Fish were anaesthetised (150 mg l^−1^ of neutralized MS222) and blood was taken via caudal puncture whilst their head was submerged and still showing signs of ventilation. Rainbow trout (*Oncorhynchus mykiss*; body mass range 506–611 g) were obtained from Hooke Springs Trout Farm, Dorset, and initially held in dechlorinated freshwater. To prepare these fish for subsequent acclimation to seawater they were fed a diet of commercial trout pellets supplemented with increasing amounts of NaCl (Perry et al. 2006; Salman and Eddy, 1990). Dietary salt content was increased in 2 % (w/w) steps every 3–4 days up to a maximum of 12 % (w/w) added NaCl. The external salinity was then gradually raised up to full-strength seawater over a 48-h period and fish were allowed to acclimate for a further 2 weeks prior to use in experiments. All experiments were conducted with the approval of the University of Exeter Ethics Committee and under a UK Home Office license (PPL 30/2217).

**Table 1:** In vivo acid–base status of mixed arterial blood from the dorsal aorta, and intestinal venous blood from the ventral intestinal vein of seawater-acclimated rainbow trout

	Dorsal aorta	Ventral intestinal vein	
pH_e_	7.93 ± 0.02	7.73 ± 0.03	(P = 0.003)*
[HCO_3_^−^] (mmol l^−1^)	11.4 ± 0.7	11.4 ± 1.0	(P = 0.456) NS
PCO_2_ (mmHg)	2.97 ± 0.10	4.68 ± 0.21	(*P* = 0.001)*

An asterisk denotes a significant difference between the ‘Dorsal aorta’ and the ‘Ventral intestinal vein’ (n = 5)

### Haemolysate preparation

Red blood cells were isolated in whole blood by centrifugation and washed three times in cold Cortland’s physiological saline (8 °C; Wolf, 1963). The red cells were lysed by addition of two times their volume of deionised water (8 °C; Millipore, Direct Q3) and subsequent freezing, and cell debris was removed by 10 min of chilled (4 °C) centrifugation at 14,000 r.p.m. (Thermo Electron Corporation 21000R, Waltham, MA, USA). To remove organic phosphates (ATP and GTP, collectively referred to as NTP) and reduce methaemoglobin levels, sodium dithionite (Sigma-Aldrich) and glycerol were added to haemolysate samples which were then run through two Sephadex PD-10 columns (GE Healthcare Bio-Sciences AB, Uppsala, Sweden) using two separate buffers (deoxygenated 50 mmol l^−1^ KCl and 50 mmol l^−1^ Hepes buffers at pH 7.5 and 8.5). To remove the Hepes buffer and further reduce NTP levels (but retain Hb) from the eluted haemolysate, each sample (~1 ml) was placed in a sealed Spectra Por molecular-porous membrane dialysis bag (molecular weight cut-off 6–8 kDa; Spectrum Medical Industries Inc. Los Angeles, USA) and left in 2-l deionised water (Milli-Q) at 5 °C for at least 12 h (deionised water was replaced three times over this period).

Haemoglobin concentration was determined after conversion to cyanomethaemoglobin using a micromolar extinction coefficient of 11 at 540 nm using the Drabkin’s reagent (Sigma-Aldrich) method (Drabkin and Austin 1935), and methaemoglobin content was assessed on identical sub-samples using the spectrophotometric method of Benesch et al. (1973) (Shimadzu UV-160 spectrophotometer).

### Haemoglobin titrations

Haemoglobin titrations were conducted according to Regan and Brauner (2010a) on concentrated stripped haemolysates that were diluted to a final concentration of 40 μmol l^−1^ of Hb tetramer (Hb_4_) and 0.1 mol l^−1^ KCl. A 2 ml volume of the haemolysate in the presence of GTP (guanosine 5’-triphosphate sodium salt hydrate, Sigma-Aldrich) at a molar ratio of 3:1 relative to the tetrameric Hb (GTP:Hb_4_) was then transferred to a chilled (12 °C), magnetically stirred glass titration vessel where the haemolysates were equilibrated with humidified oxygen (100 %) for 90 min. A 3:1 GTP:Hb_4_ ratio was chosen to ensure all Hb molecules came under the allosteric influence of GTP (Regan and Brauner 2010a). Although this is higher than the measured ratio of 1.63:1 found in flounder RBCs (Fletcher 1975; Val 2000), experiments by Cooper et al. (unpublished) and Pelster and Weber (1990) suggest that this difference would not affect the Hb buffer capacity or Root effect. The buffer value of GTP at this concentration was subtracted from the Hb buffer values. Hydrogen ion titrations were performed with an automated Radiometer TitraLab 90 titration apparatus (Copenhagen, Denmark), where 0.01 mol l^−1^ NaOH (‘Baker analysed’; J.T. Baker) was added in 10-μl increments to raise pH from iso-ionic to 9.2. After 5 min of equilibration at a pH 9.2, titration with 0.01 mol l^−1^ HCl (10 μl increments; ‘Baker analysed’; J.T. Baker) was initiated and continued until pH 5.2 was reached. The total amount of NaOH or HCl required to reach these endpoints was recorded. The same procedure was performed on a separate 2 ml sample from the same stock haemolysate, this time equilibrated in humidified nitrogen (100 %) to deoxygenate the Hb molecules.

The resulting titration curves were used to calculate Hb buffer values and Haldane effects. The negative slope of a titration curve at a particular pH is proportional to the Hb buffer value at that pH. Thus, buffer values between pH 5 and 9 were determined for both oxygenated and deoxygenated Hbs by calculating the negative slopes between adjacent data points on each of the oxygenated and deoxygenated Hb titration curves. Fixed acid Haldane effects were determined by calculating the vertical distances between their oxygenated and deoxygenated Hb titration curves (δZ_H_, mol H^+^ taken up per mol Hb_4_ upon deoxygenation at constant pH).

### Root effect analysis

The magnitude of the Root effect over a broad pH range was determined according to Regan and Brauner (2010b) by measuring oxygen saturation of Hb spectrophotometrically at atmospheric *P*O_2_ (157 mmHg) in Tris buffers (50 mmol l^−1^; Trizma hydrochloride, Sigma-Aldrich) ranging in pH from 5.5 to 8.5. Air-equilibrated concentrated haemolysates were then mixed with the buffers in a 1-ml cuvette and diluted to a final concentration of 160 mmol l^−1^ Hb_4_ and 0.1 mol l^-1^ KCl. This procedure was conducted for haemolysates in the presence of GTP (GTP:Hb_4_ ratio of 3:1). Absorption at wavelengths of 540, 560 and 576 nm were measured and recorded using a Shimadzu UV-160 spectrophotometer, and were used to calculate percent Hb-O_2_ saturation according to the following equations from Benesch et al. (1973):

1 $$[ {\text{Oxy Hb]}} = ( 1. 4 7 4 7 {\text{ A}}_{ 5 7 6}\, {-}\,0. 6 8 20{\text{A}}_{ 5 60} {-}0. 5 3 2 9 {\text{ A}}_{ 5 40} )$$

2 $$[ {\text{Deoxy Hb]}} = ( 1. 4 7 4 9 {\text{A}}_{ 5 60} + 0. 2 1 4 1 {\text{A}}_{ 5 7 6} - 1. 10 4 2 {\text{A}}_{ 5 40} )$$

Where A is the absorbance at either 540, 560 or 576 nm. Eq. (1) and (2) were then added together to give total Hb in solution, by which the oxygenated Hb concentration was divided to yield the percent oxygenation status of the haemolysate at each pH.

### Estimating the blood acidosis within intestinal capillaries

We calculated the reduction in blood pH in the capillary of the intestine that would be expected due to intestinal HCO_3_^−^ secretion using the following assumptions for flounder in normal strength to triple strength seawater (i.e. salinities of 33 to 90):

1. The non-bicarbonate buffer capacity in whole blood is proportional to the haematocrit value and can be derived from the linear regression slope of the [HCO_3_^−^] vs. pH relationship, which has been calculated for starry flounder (*Platichthys stellatus*) as

3 $$\beta = - 1 6. 5\times {\text{Hct}} \cdot {-}\; 2. 8 9$$

where *β* is the buffering capacity (expressed in slykes; mmol l^−1^ pH^−1^) and Hct· is the haematocrit expressed as a fraction (Wood et al. 1982). Therefore, at haematocrits of 15–20 %, the non-bicarbonate buffer capacity would equate to approximately −5 to −6 mmol l^−1^ pH^−1^.

2. The expected change in [H^+^] (δ [H^+^], mEq l^−1^) in the intestinal venous capillaries was calculated as:

4 $${\delta [}{\text{H}}^{ + } \text{]} = [ {\text{H}}^{ + } ] {\text{ loading}}\,{\text{rate}}\,{\text{into}}\,{\text{the}}\,{\text{blood}}\,/\,{\text{blood}}\,{\text{flow}}\,{\text{rate}}$$

where the potential [H^+^] loading rate into the blood (250–500 μEq kg^−1^ h^−1^) was divided by blood flow rate (ranging from 246 to 828 ml kg^−1^ h^−1^) (red Irish lord, *Hemilepidotus hemilepidotus*, Axelsson et al. 2000; European sea bass, *Dicentrarchus labrax*, Altimiras et al. 2008). It is important to note that intestinal blood flow rates do not exist for the species of interest. In the aforementioned studies, ultrasonic flow probes were implanted on the ventral aorta and the gastrointestinal arteries. The blood flow in the intestinal capillaries would be much lower than these, thus our estimates are extremely conservative. Potential blood [H^+^] loading data were taken from Whittamore et al. (2010), where Ca^2+^ was perfused into the intestine of flounder simulating the ingestion of seawater at salinities of 33 to 90. Incorporating the above range of blood flow rates and potential [H^+^] loading, a δ [H^+^] of between 0.3 and 2.0 mEq l^−1^ was predicted.

3. To calculate the change in blood pH (δ pH) in the intestinal capillaries, the following equation was used:

5 $$\updelta\,{\text{pH }} = \left( {\updelta\,[{\text{H}}^{ + } ] + \left( {[{\text{HCO}}_{ 3}^{ - } ]_{\text{Arterial}} - [{\text{HCO}}_{ 3}^{ - } ]_{\text{Venous}} } \right)} \right)/\beta$$

Which accounts for the predicted H^+^ loading in the intestinal capillaries (δ [H^+^]), the non-bicarbonate buffer capacity (*β*), and the typical systemic difference in [HCO_3_^−^] between venous (7.12 ± 0.32 mmol l^−1^; *n* = 5) and arterial (6.12 ± 0.37 mmol l^−1^; *n* = 4]) blood from cannulated flounder (Cooper, Whittamore and Wilson, unpublished data).

4. Finally, using the following equation we converted our extracellular pH values (pH_e_) to red blood cell (RBC) intracellular pH (pH_i_):

6 $${\text{pH}}_{\text{i}} = 0. 5 9\times {\text{pH}}_{\text{e}} + 2. 7 1$$

Using constants based on data from rainbow trout at 10 °C (Heming et al. 1986).

### In vivo analysis of intestinal blood acid–base balance in trout

It would be ideal to compare the above estimations of blood pH reduction within intestinal veins with those actually found in vivo in European flounder. However, in preliminary trials, it proved impossible to successfully cannulate the intestinal vein of flounder due to the complex vasculature, so we resorted to using seawater-acclimated rainbow trout as a pragmatic alternative. Seawater-acclimated rainbow trout (*n* = 5) were anaesthetised with MS222 (100 mg l^−1^ buffered with 200 mg l^−1^ NaHCO_3_, followed by prolonged aeration) and transferred to a surgery table where their gills were irrigated with temperature-controlled (14 °C) seawater containing a lower dose of buffered MS222 (60 mg l^−1^). The dorsal aorta was cannulated as previously described by Cooper and Wilson (2008), to provide a source of arterial blood that reflects that supplying the intestine. The ventral intestinal vein was then cannulated near the proximal end of the posterior intestine. Cannulae for the ventral intestinal vein were prepared similarly to those used for the hepatic portal vein cannulation as described by Eliason et al. (2007), with a 15-mm tip made of silastic (ID = 0.5 mm, OD = 0.94 mm) stretched over the end of PE50 tubing with a small bubble created ~3 mm from the end. A local anaesthetic (Lidocaine, 20 mg ml^−1^; Centaur Services, UK) was used to prepare the laparotomy site by subdermal injections (8 × 0.05 ml) along the incision line, running parallel with the lateral line from just above to slightly posterior to the right pelvic fin. A scalpel was then used to make a ~2.5 cm skin incision, and the underlying muscle was separated by scalpel and blunt dissection, and finally held open using retractors. The intestinal vein has two branches running along the dorsal and ventral sides of the intestine, connected at regular intervals by many circumferential veins that encircle the intestine (Olson, 2000). The ventral intestinal vein was located and two pieces of silk suture (4–0) were threaded between the vein and the underlying intestine about 5–8 mm apart. The posterior thread was tied to occlude the vein, and the anterior thread was loosely knotted leaving the vessel unoccluded. A small cut was then made in the vein using micro-scissors in between the two silk threads and the silastic tip of the venous cannula quickly inserted into the vein. The cannula tip was pushed forward about 15 mm into the vein and the anterior thread secured tightly around the vessel and cannula adjacent to the bubble (Eliason et al. 2007). The cannula was then flushed with a sterile solution of 150 mmol l^−1^ NaCl containing sodium heparin (150 I.U. ml^−1^). The skin incision was then closed using continuous silk sutures (2–0) and the cannula was secured to the skin at two positions, the first ~5 mm posterior to the skin incision site, and the second on the dorsal surface near the adipose fin. The skin at the incision site was then re-coated with mucus from a nearby but untreated area of skin, and the fish was recovered in anaesthetic-free seawater. Fish were closely monitored for the first 10 min during which time normal ventilation and self-righting reflexes were re-established, and then left for 24 h to recover from the surgery.

Blood samples were taken from the dorsal aorta and the ventral intestinal vein and then analysed for whole blood pH, plasma total CO_2_ (*T*CO_2_) and haematocrit, as described in Cooper and Wilson (2008). Plasma *P*CO_2_ and [HCO_3_^−^] were calculated from plasma *T*CO_2_ and blood pH measurements using a rearrangement of the Henderson–Hasselbalch equation and values for solubility (αCO_2_ = 0.064 mmol l^−1^ mmHg^−1^) and pK_app_ (6.11–6.17—based on measured temperature and pH), according to Boutilier et al. (1984). An estimate of the metabolic acid addition to the blood (δH_m_^+^) during transit from arterial to venous vessels was calculated according to McDonald et al. (1980):

7 $$\updelta\,{\text{H}}^{ + }_{\text{m}} = [{\text{HCO}}_{ 3}^{ - } ]_{\text{Arterial}} - [{\text{HCO}}_{ 3}^{ - } ]_{\text{Venous}} {-}\left( {\beta \, \times ({\text{pH}}_{\text{Arterial}} {-}{\text{pH}}_{\text{Venous}} )} \right)$$

where (similar to Eq. 3 for flounder), non-bicarbonate buffer values (*β*) were estimated from the blood haematocrit values expressed as a decimal (Hct·) using the regression relationship of Wood et al. (1982) established for rainbow trout whole blood:

8 $$\beta = ( - 2 8. 3 5\times {\text{Hct}} \cdot ){-} 2. 5 9$$

### Statistics

Blood acid–base variables for the paired arterial and venous sources of the same individual fish were compared using a Student’s paired *t* test. This included blood from the dorsal aorta and ventral intestinal vein from rainbow trout in the present study (see Table 1), as well as blood from the dorsal aorta and ventral aorta (or from an equivalent mixed venous source) from 12 previously published studies on rainbow trout (see Table 2). Means were considered significantly different when *P* < 0.05.

**Table 2:** In vivo acid–base status of mixed arterial and mixed venous blood of freshwater-acclimated rainbow trout

	Mixed arterial	Mixed venous	
pH_e_	7.87 ± 0.03	7.86 ± 0.03	(P = 0.091)
[HCO_3_^−^] (mmol l^−1^)	7.8 ± 0.9	9.8 ± 0.9	(P = 0.008)*
*P*CO_2_ (mmHg)	2.16 ± 0.19	2.70 ± 0.27	(*P* = 0.011)*

Blood pH data were extracted from 12 different previously published data sets in which individual fish had cannulae implanted in the dorsal aorta and ventral aorta or a comparable source of mixed venous blood (Brauner et al. 2000; Cameron and Heisler, 1983; Currie and Tufts 1997; Holeton and Randall, 1967; Kiceniuk and Jones, 1977; Milligan and Wood, 1986; Nikinmaa and Vihersaari, 1993; Perry et al. 1997; Soivio et al. 1981; Thomas et al. 1994). Data on blood [HCO_3_^−^] and PCO_2_ were only available from four of these previously published data sets

An asterisk denotes a significant difference between ‘Mixed arterial’ and ‘Mixed venous’

## Results

### Haemoglobin titrations and buffer values

**Fig. 1:**
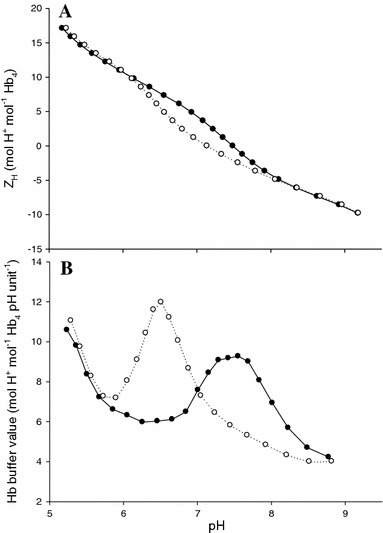
Representative H^+^ titration and buffer value curves for European flounder haemoglobin. Panel A: Hb-H^+^ titration curves, *Z*_H_ (mol H^+^ mol^−1^ Hb_4_) as a function of pH, for oxygenated (*open symbols*) and deoxygenated (*filled symbols*) Hb solutions. Panel B: Hb buffer values (−δ*Z*_H_/δpH) as a function of pH for oxygenated (*open symbols*) and deoxygenated (*filled symbols*) Hb solutions. A total of nine titrations were performed on stripped haemolysates at a [Hb_4_] of 0.04 mmol l^−1^ and a [KCl] of 0.1 mol l^−1^, in the presence of organic phosphates (3:1 ratio of GTP:Hb_4_)

Representative H^+^ titration curves of the oxygenated and deoxygenated Hbs from European flounder show how net proton charge (Z_H_, mol H^+^ mol^−1^ Hb_4_) of stripped Hbs in 0.1 mol l^−1^ KCl changes as a function of pH in the presence of GTP (3:1 molar ratio of GTP:Hb_4_; Fig. 1a). Zero net proton charge refers to the isoelectric pH and is used as the reference point (Tanford, 1962). Titration curves were unaffected by the different acclimation salinities.

The slope of the Hb titration curve at a particular pH is representative of the Hb buffer value at that pH (mol H^+^ mol^−1^ Hb_4_ pH unit^−1^). The oxygenated Hbs of flounder exhibit a maximum buffer capacity of ~12 mol H^+^ mol^−1^ Hb_4_ pH unit^−1^ at pH 6.5, while the deoxygenated Hbs show a lower buffer value (9 mol H^+^ mol^−1^ Hb_4_ pH unit^−1^) that peaked at a higher pH (7.5; Fig. 1b). These trends are similar to the Hb-H^+^ binding characteristics of other teleosts measured to date.

### The Haldane effect

**Fig. 2:**
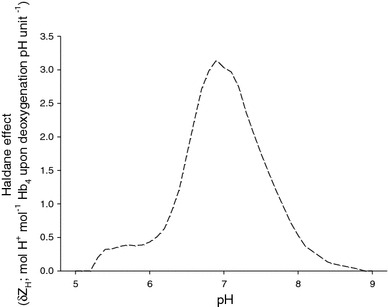
Fixed-acid Haldane effect (δ*Z*_H_; number of protons taken up per Hb_4_ upon deoxygenation at constant pH) as a function of pH in European flounder, calculated from the vertical distance between the oxygenated and deoxygenated titration curves (Fig. 1)

The vertical distance, or δZ_H_, between the oxygenated and deoxygenated titration curves indicates the fixed acid Haldane effect (δZ_H_, mol H^+^ taken up per mol Hb_4_ upon deoxygenation at constant pH), which varies according to pH (Fig. 2). A maximum of ~3.2 H^+^ were taken up (by each Hb tetramer) upon deoxygenation at pH 7.0 in the presence of GTP (3:1 molar ratio of GTP:Hb_4_) in flounder (Fig. 2).

### The Root effect

**Fig. 3:**
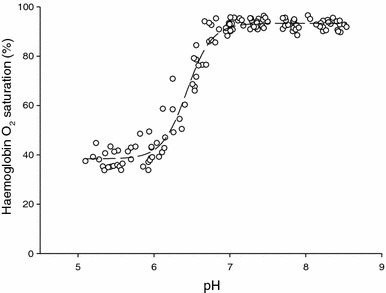
Root effect of European flounder haemoglobin determined spectrophotometrically in the presence of organic phosphates (3:1 ratio of GTP:Hb_4_). Correlation coefficient of fitted line: *R* ^2^ = 0.968

Analysis of flounder Hbs revealed a significant Root effect in the presence of GTP, with an onset pH value of ~6.9 and a maximal Hb-O_2_ desaturation of ~55 % occurring at pH values of 6.0 and lower (Fig. 3). The Root effect properties were unaffected by the different acclimation salinities.

### Modelling of the blood acidosis within intestinal capillaries

**Fig. 4:**
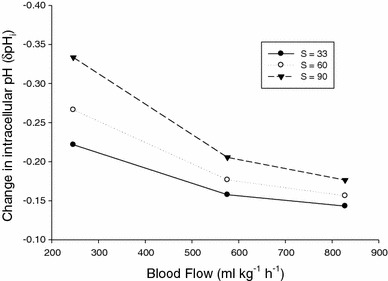
The effects of different blood flow rates and salinities on red blood cell intracellular pH values (δpH_i_) in European flounder. Blood flow rates range from 246 to 828 ml kg^−1^ h^−1^, H^+^ loading into the blood ranges from 250 to 500 μEq kg^−1^ h^−1^, and imbibed seawater is simulated at a salinity (S) = 33(*filled circles*), = 60 (*open circles*) and = 90 (*inverted triangles*). See Eq. 3–6 for details

Our calculations (Eq. 3–6) predicted a RBC pH_i_ reduction in the range 0.14 pH units (relatively high blood flow at the lowest salinity of 33) to 0.33 pH units (relatively low blood flow at the highest ambient salinity of 90) (Fig. 4). Assuming an arterial whole blood pH in flounder of 7.90 (Cooper et al. 2010), this would equate to an arterial pH_i_ of 7.37. By contrast, we would predict Hbs of flounder to be exposed to pH_i_ values from 7.37 to 7.04 in the capillaries supplying the intestinal cells when fish are living in salinities ranging from 33 to 90 (and intestinal blood flow rates ranging from 246 to 828 ml kg^−1^ h^−1^; Fig. 4).

### In vivo blood acid–base status of arterial and intestinal vein in seawater trout

Blood from the ventral intestinal vein of seawater-acclimated trout was 0.20 pH(_e_) units lower on average compared to mixed arterial blood from the dorsal aorta of the same fish (Table 1). Using Eq. 6 (above), a pH_e_ drop of 0.20 units translates to a RBC pH_i_ decrease of 0.12 units. In contrast there was no significant difference in pH when comparing blood from mixed arterial and mixed venous sources in cannulated freshwater-acclimated rainbow trout from twelve previously published data sets (Table 2). The [HCO_3_^−^] and *P*CO_2_ of mixed venous blood were both 25 % higher than that of mixed arterial blood (Table 2), whereas [HCO_3_^−^] was unchanged but *P*CO_2_ was 58 % higher in the ventral intestinal vein (present study) compared to dorsal aorta (Table 1). Calculations based on Table 1 resulted in a metabolic acid load (δH_m_^+^) of +1.9 mmol l^−1^ being added to the blood during transit through the vasculature of the posterior intestine of seawater trout, whereas a negative value of −1.8 mmol l^−1^ was calculated for δH_m_^+^ when comparing mixed venous blood (either ventral aorta or Ductus of Cuvier) with the same arterial source (dorsal aorta) in freshwater-acclimated rainbow trout (Currie and Tufts 1997; Milligan and Wood 1986; Nikinmaa and Vihersaari, 1993). This represents a significantly greater net acid load of almost 4 mmol l^−1^ within the intestinal veins of seawater trout when compared to the average mixed venous blood.

## Discussion

**Fig. 5:**
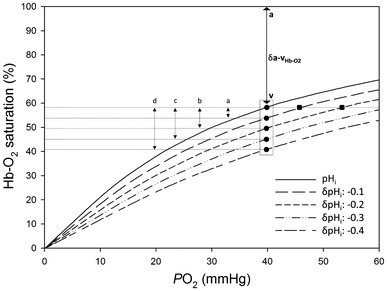
Oxygen equilibrium curves (OECs) for European flounder haemoglobin modelled using the Hill equation. Solid curve represents resting red blood cell (RBC) pH_i_ of 7.37 and is based on a Hb P_50_ value of 30 mmHg (Jensen et al. 2002) and a Hill coefficient of 1.2 (Jensen et al. 2002), while hashed curves represent those OECs at RBC pH_i_ values below resting levels. Filled circles enclosed in box depict differences in Hb-O_2_ at a *P*O_2_ approximating venous levels (40 mmHg, based on Cooper et al. 2010) as a function of pH_i_, with lower case letters showing differences in Hb-O_2_ saturation that come with a, *a* 0.1, *b* 0.2, *c* 0.3 and *d* 0.4 reduction in RBC pH_i_ below resting levels. Bolded “a” at top of figure represents arterial Hb-O_2_ saturation percentage of 100 %, while bolded “v” represents venous Hb-O_2_ saturation percentage of 58 % (based on the aforementioned 40 mmHg venous *P*O_2_). Filled squares represent the *P*O_2_ changes that result from a 0.1 and 0.2 reduction in RBC pH_i_ at a constant Hb-O_2_ saturation percentage of 58 % (see discussion for details)

Through the analysis of H^+^ equilibria and gas exchange properties of European flounder (*Platichthys flesus*) Hb,  we discuss how these pH-dependent Hb characteristics may be associated with enhanced O_2_ delivery to supply the increased metabolic demands of the intestine during the osmoregulatory work associated with drinking seawater in high salinities in marine teleosts. According to our model, osmoregulatory processes could result in intestinal capillary pH_i_ ranging from 7.37 to 7.04 (Eqs. 3–6), placing RBC pH_i_ directly on the “shoulder” of the Root effect curve (Fig. 3). While this means that O_2_ delivery may not be enhanced via the Root effect during osmoregulation in this species, this pH_i_ change (Fig. 4) is sufficient to exploit flounder’s Bohr effect and enhance intestinal O_2_ delivery by up to 42 % with no change to tissue blood flow (Fig. 5). While this phenomenon has yet to be quantified directly in vivo, it is likely applicable to all teleost fish in hyperosmotic environments.

### The potential for enhanced O_2_ delivery to the intestinal cells via the Bohr effect

The degree to which O_2_ delivery to intestinal cells could be enhanced is a product of the RBC pH_i_ change during blood capillary transit and the intracellular Bohr coefficient. While the Haldane effect describes the change in Hb-H^+^ affinity that results from a change in Hb-O_2_ saturation, the Bohr effect describes a change in Hb-O_2_ affinity that results from a change in pH. These two phenomena are thermodynamically linked at the molecular level (Wyman, 1973), and this linkage is illustrated by the equation:

9 $$- \delta \,{ \log }P_{ 50} /\delta{\text{pH}} = \delta{\text{H}}^{ + }$$

where δH^+^ is the number of moles of protons released per mole of O_2_ bound to Hb (i.e. the Haldane coefficient, derived from Fig. 2), and the Bohr coefficient is the numerical negative of this value (Jensen and Weber, 1985). At a pH_i_ of 7.37, the Hb of European flounder releases 2.52 mol of protons for every 4 mol of O_2_ bound, resulting in a Haldane coefficient of 0.63 and thus a Bohr coefficient of −0.63. Assuming a blood *P*_50_ (*P*O_2_ at which Hb is 50 % saturated) value of 30 mmHg (Jensen et al. 2002), changes in *P*_50_ can be estimated over the calculated range of RBC pH_i_ values within intestinal capillaries determined at the respective salinity and intestinal blood flow rates given above (Eq. 3–6; Fig. 4). Oxygen equilibrium curves (OECs) can then be constructed from these δ*P*_50_ values by re-arranging the Hill equation (Hill, 1910):

10 $${ \log }\,P{\text{O}}_{ 2} = \, \left( {\left( {{ \log }\,(y/ 1- y)} \right)/n} \right) + { \log }\,P_{ 50}$$

where *y* is Hb-O_2_ saturation and *n* is the Hill coefficient of European flounder (1.2, from Jensen et al. 2002). The resulting curves are depicted in Fig. 5, where the solid line represents the OEC at control values for RBC pH_i_, and the four hashed lines beneath it represent calculated OECs at progressively lower RBC pH_i_ values.

The degree to which the calculated RBC pH_i_ changes could enhance O_2_ delivery can be interpolated directly from these OECs. Assuming that arterial blood saturation is always close to 100 % and venous *P*O_2_ (*P*_v_O_2_) is 40 mmHg (Cooper et al. 2010), the a-v Hb-O_2_ extraction with no change in RBC pH_i_ (Fig. 5 solid line) would be 42 % (i.e. 100 − 58 %). The calculated reductions in RBC pH_i_ result in a large increase in *P*_v_O_2_ with constant a-v Hb-O_2_ extraction (i.e. trace horizontally across the curves from the point mentioned above), increasing the driving force for O_2_ delivery. For example, *P*_v_O_2_ would be increased from 40 mmHg to 46 or 53.5 mmHg with a 0.1 or 0.2 pH_i_ unit reduction, respectively. Assuming a constant *P*_v_O_2_ (whereby enhanced metabolism by the tissues instantly draws the “extra” O_2_ available from the blood), the calculated reductions in RBC pH_i_ could have a large effect on a-v Hb-O_2_ saturation, thus enhancing delivery. Again assuming arterial saturation remains close to 100 %, a drop in RBC pH_i_ of 0.1 units would increase a-v Hb-O_2_ extraction to 45 % (point a, Fig 5; 100 − 55 %), which could represent a 7 % increase in O_2_ delivery relative to the control (45/42 × 100 = 107 %) with no change in blood flow or *P*_v_O_2_. A similar analysis for a RBC pH_i_ reductions of 0.2, 0.3 and 0.4 units yields an enhanced O_2_ delivery relative to the control of 20 % (point b, Fig 5; 50.5/42 × 100 = 120 %), 31 % (point c, Fig 5; 55/42 × 100 = 131 %) and 42 % (point d, Fig 5; 59.5/42 × 100 = 142 %), respectively, indicating that this system has a tremendous potential to facilitate O_2_ delivery to this metabolically active tissue.

The RBC pH_i_ reductions discussed above were calculated instead of being measured because the cannulation of flounder’s intestinal vein proved impossible and, to our knowledge, no other direct measurements from the intestinal capillaries of flounder or any other species have been made. However, we were able to successfully cannulate the ventral intestinal vein of seawater-acclimated rainbow trout. In vivo pH_e_ measurements were consistently 0.2 pH units lower than mixed arterial blood from the dorsal aorta (Table 1), equating to a pH_i_ decrease of 0.12 pH units (Eq. 6). This difference in blood pH is likely directly associated with osmoregulation in a marine environment, as the pH differences between mixed arterial and mixed venous sources of blood are minimal or in some cases even reversed (Brauner et al. 2000a, b; and see Table 2). These in vivo measurements match the predictions made by our model, when using trout-specific data, remarkably well. For example, when using a non-bicarbonate buffer (*β*) value of −9.48 calculated for rainbow trout whole blood (Wood et al. 1982) and measured values for arterial pH_e_ (7.93; Table 1), arterial and venous HCO_3_^−^ concentration (11.40 mmol l^−1^; Table 1), and H^+^ loading into the venous blood (1.9 mmol l^−1^; dorsal aorta vs. ventral intestinal vein), the pH_i_ prediction of our rainbow trout model is consistent with the measured values (i.e. a reduction of 0.12 pH_i_ units; Eq. 5 and 6). Taking these calculations forward, again using trout-specific data, trout OECs can be produced and then compared to those from flounder. Using an intracellular trout Bohr coefficient of −0.70 (Jensen, 1989), a *P*_50_ value of 23 mmHg (Tetens and Christensen, 1987), a *P*_v_O_2_ of 55 mmHg (Brauner et al. 2000a), and assuming that arterial blood saturation is always close to 100 %, our trout OECs predict that a drop in RBC pH_i_ of 0.1 units would represent a 18 % increase in O_2_ delivery relative to the control (Eq. 10). This is similar to what was calculated in the flounder model (i.e. a 0.1 pH_i_ decrease equating to a 7 % increase in O_2_ delivery relative to the control; Fig. 5). It is worth noting that predictions for a pH_i_ decrease of 0.1 units in both species are based on these fish osmoregulating in normal strength seawater. Our flounder model goes a step further and uses H^+^ loading data from fish osmoregulating in double and triple strength seawater. Although extreme, this model demonstrates the potential for osmoregulation in hyperosmotic environments to directly enhance O_2_ delivery to the enterocytes.

The measured pH reduction in vivo is physiologically significant and very close to our calculated predicted change in blood pH. Importantly, these predictions are likely an underestimate of the most extreme potential pH change occurring in the blood supplying HCO_3_^−^-secreting enterocytes for two reasons. Firstly, the intestinal vein we cannulated collects venous blood that drains from all the tissues of the intestine, the major volume of which will be the submucosa, and the circular and longitudinal smooth muscle layers, whereas the HCO_3_^−^-secreting epithelium adjacent to the gut lumen will make up the minor portion. Secondly, the highest rates of intestinal HCO_3_^−^ secretion are likely to be from those cells located in the anterior region, where the ingested Ca^2+^ ions that drive HCO_3_^−^ secretion first enter the intestine (Wilson et al. 2002). By contrast, we cannulated the ventral intestinal vein at the posterior end of the intestine. Attempts were made to directly access the venous blood draining the anterior section of the intestine, but this proved to be impossible due to the location of the pyloric caeca in rainbow trout. Nevertheless, this conservative measurement of the arterial–venous blood pH difference is still very large (Fig. 5), and one that is certainly capable of increasing the O_2_ delivery potential of H^+^ sensitive Hbs.

This level of enhanced O_2_ delivery may be important to satisfy the metabolic demands of intestinal tissue. European flounder acclimated to double strength seawater (salinity = 60) were shown to have a 50 % higher O_2_ consumption rate at the whole body level than those acclimated to a salinity of 33 (2.25 vs. 1.5 mmol kg^−1^ h^−1^; Cooper and Wilson, unpublished). It is not known whether these whole body metabolic changes are representative of those that would occur at the intestinal epithelium, however, using isolated intestinal tissue in a specialized in vitro Ussing chamber set-up, Taylor and Grosell (2009) showed that following a meal, O_2_ consumption and HCO_3_^−^ secretion increased 1.9- and 1.6-fold, respectively. These data highlight how metabolically demanding HCO_3_^−^ secretion can be, both in vivo and in vitro. Any increase in intestinal *P*O_2_ initiated by the Bohr effect would therefore be of major benefit to the fish.

Finally, it is also relevant to note that the total CO_2_ (11.70 ± 1.04 mmol l^−1^) in the intestinal vein of rainbow trout was not significantly different from that in the arterial supply (11.54 ± 0.73 mmol l^−1^) to this tissue (reflected in the data for plasma [HCO_3_^−^] in Table 1), whereas mixed venous blood typically has a total CO_2_ (and [HCO_3_^−^]) that is 2 mmol l^−1^ or about 25 % higher than mixed arterial blood (Table 2). This suggests there was zero net excretion of respiratory CO_2_ from the intestine into the blood. Taken at face value, this suggests that the demand for apical HCO_3_^−^ secretion was equivalent to the entire respiratory CO_2_/HCO_3_^−^ produced by all the tissues supplied by the intestinal vasculature (i.e. the epithelium plus underlying submucosa, and circular and longitudinal smooth muscle layers etc.). It therefore seems likely that apical HCO_3_^−^ secretion by the enterocytes cannot be fuelled by their endogenous respiratory CO_2_ production alone, even under the relatively mild hyperosmotic conditions of normal seawater. This situation will be even more extreme under hypersaline conditions—the intestine is likely to become a net consumer of both CO_2_ and O_2_ from its vascular blood supply, rather than driving a roughly equal exchange of these two respiratory gases as is the norm in animal tissues.

### Additional mechanisms for enhanced O_2_ delivery to the intestine

The teleost eye and swim bladder have long been posited as the only tissues in the body capable of generating sufficient blood acidoses to activate the Root effect and greatly enhance oxygen delivery. But recent work suggests that a mild acidosis in the presence of plasma-accessible carbonic anhydrase (CA) may greatly enhance oxygen delivery in muscle too. In a closed in vitro system, the addition of plasma-accessible CA to acidified rainbow trout RBCs has been shown to short circuit pH regulation via Na^+^/H^+^ exchange (NHE), decreasing Hb-O_2_ saturation and elevating blood *P*O_2_ by ~30 mmHg (Rummer and Brauner, 2011). In vivo, plasma-accessible CA in the presence of a mild acidosis has been estimated to double muscle oxygen delivery with no change in blood flow (Rummer et al. 2013). Our model for the intestine does not take into account the potential effect of plasma-accessible CA on RBC pH_i_. However, in the enterocytes of teleost fishes, various CA isoforms are present on the apical membrane and in the cytosol (Gilmour et al. 2012; Grosell et al. 2007; Sattin et al. 2010) similar to the muscle (Wang et al. 1998; Henry and Swenson, 2000) and the arterial blood supply (Randall et al. 2014), and potentially in the basolateral extracellular fluid (although this has yet to be characterized, Taylor et al. 2011). It is therefore likely that RBCs in close proximity to the intestine will come into contact with CA. Exposing the intestine to hypersaline conditions results in a mild systemic-wide blood acidosis (Cooper et al. 2010), which in the presence of plasma-accessible CA, could be transmitted to the RBC, further reducing pH_i_ from those calculated here. If operational, our pH_i_ calculations could be a considerable underestimation, and O_2_ delivery may be enhanced to an even greater degree than that proposed here owing to a potential Root effect-activating blood acidosis (i.e. pH 6.9; Fig. 3). Further studies at the capillary level will be necessary to elucidate the role of plasma-accessible CA in maximizing O_2_ delivery to the intestinal cells. Plasma-accessible CA could also hasten the RBC acidosis, which could be important if capillary transit times through the intestine are very short. We are currently pursuing further studies at the capillary level to determine transit times, confirm the presence of plasma-accessible CA at the intestine and to evaluate its potentially crucial role in maximizing O_2_ delivery to the intestinal cells.

### Concluding remarks

We have shown that the marine teleost intestine may take advantage of the unique pH-dependent properties of teleost Hb to greatly enhance O_2_ delivery to the intestinal cells that have constitutively high-energy demands associated with their osmoregulatory role in hyperosmotic environments. It was hypothesized that the secretion of HCO_3_^−^ into the intestinal lumen that results in an equivalent transfer of protons into the blood supply would decrease RBC pH_i_ sufficiently to exploit the Bohr and possibly Root effects, enhancing O_2_ delivery. Our analysis of European flounder Hb properties allowed us to construct a model that suggests this is indeed the case. Furthermore, when trout-specific values were used for the model’s variables, the resulting predictions are consistent with the measured in vivo pH difference between arterial and venous blood serving the intestine of seawater-acclimated rainbow trout, lending further support to the model. An extremely conservative estimate suggests a 0.1 unit pH change in the intestinal blood supply would enhance O_2_ delivery to enterocytes by 7 %. A more realistic estimation of intestinal blood pH change suggests this number is no less than 25 % in fish at normal marine salinities. If plasma-accessible carbonic anhydrase is present at the intestinal cell, then RBC pH_i_ could be even further reduced, with correspondingly greater increases in O_2_ delivery. To accurately confirm this, micro-scale pH measurements within intestinal capillaries are required, and thus warrants further investigation.

Intriguingly, the system we propose would serve as an elegant example of autoregulation linking osmoregulation and gas transport to satisfy the energetic costs of hypo-osmoregulation in teleosts. In essence, the greater the demand for metabolically sourced HCO_3_^−^ resulting from increased salinity and drinking rate, the greater the enhancement of O_2_ delivery to the intestine via a localized acidosis and a pH-sensitive Hb. This would operate in all marine teleosts, but such autoregulation may be especially important for fish living in variable salinity environments, and in particular, hypersaline environments. A suitable species with respect to the “Krogh Principle” for studying this phenomenon would be the Arabian killifish (*Aphanius dispar*), a remarkable animal capable of living in salinities five times greater than seawater (Plaut, 2000). In any case, our results suggest that the marine teleost intestine may utilize a pH-dependent mechanism of enhanced O_2_ delivery. This autoregulation would specifically support the osmoregulatory component of this truly multifunctional organ in fish, adding to the intrigue of its various roles in digestion, nutrient absorption, excretion, acid–base regulation and gas exchange.
